# Combining separate wound care services into an integrated whole. The integrated complex wound care team in Greenwich

**DOI:** 10.1186/1757-1146-3-S1-P2

**Published:** 2010-12-20

**Authors:** Alison Beasley, Kate Forsyth, Kim Socrates, Sonia Kirwin, Christine Kenney, Anita Murray, Suzy Taylor

**Affiliations:** 1 Greenwich Community Health Services, London, UK

## 

Definition: Integration = *an act or instance of combining into a complete whole*.

The Integrated Complex Wound Care Team comprises: the Tissue Viability Service, The Lower Limb Service and the Foot Health / Podiatry Wound Care Team.

Each specific, detached service consists of skilled, highly trained health professionals who provide expertise and management of patients with wounds:

**○ The Tissue Viability Service** provides advice, support and training to those managing patients with complex wounds. This includes maintaining skin integrity wherever possible, promoting healing in the non-healing wound and provision of expertise and specialist equipment to complex acute and chronic wounds.

**○ The Podiatry Wound Care Service** is a team of experienced podiatrists highly skilled to manage the high risk foot. The team works across both primary and secondary care forming a seamless service between the two working closely with all disciplines to provide a holistic approach to treatment and care planning.

**○ The Lower Limb Service** is run by experienced nurses who are proficient in the management of cellulitis, tissue viability and skin care related to the lower limb. Patients are provided with a variety of health education, clinical treatment and advice in different settings including community clinics, domiciliary visits and groups within the wider community.

Each service has a different view of the patient and their wound.

Working within an integrated team allows us to fast track urgent referrals and arrange joint visits between the disciplines with ease so wound care plans can be devised and implemented immediately. The 3 services complement each other well, each bringing a different skill mix to the treatment but with us all having the same goal

Just as we view the patient holistically but assess individual systems, so the Integrated Wound Care Team is able to take a different view of the wound but collaborate together to provide high quality care which benefits the patient as a whole.

